# Evolutionary and Biotechnological Implications of Robust Hydrogenase Activity in Halophilic Strains of Tetraselmis

**DOI:** 10.1371/journal.pone.0085812

**Published:** 2014-01-21

**Authors:** Sarah D'Adamo, Robert E. Jinkerson, Eric S. Boyd, Susan L. Brown, Bonnie K. Baxter, John W. Peters, Matthew C. Posewitz

**Affiliations:** 1 Department of Chemistry and Geochemistry, Colorado School of Mines, Golden, Colorado, United States of America; 2 Department of Microbiology and the Thermal Biology Institute, Montana State University, Bozeman, Montana, United States of America; 3 Center for Marine Microbial Ecology and Diversity, University of Hawaii, Honolulu, Hawaii, United States of America; 4 Department of Biology and the Great Salt Lake Institute, Westminster College, Salt Lake City, Utah, United States of America; 5 Department of Chemistry and Biochemistry, Montana State University, Bozeman, Montana, United States of America; Universidade Federal de Vicosa, Brazil

## Abstract

Although significant advances in H_2_ photoproduction have recently been realized in fresh water algae (e.g. Chlamydomonas reinhardtii), relatively few studies have focused on H_2_ production and hydrogenase adaptations in marine or halophilic algae. Salt water organisms likely offer several advantages for biotechnological H_2_ production due to the global abundance of salt water, decreased H_2_ and O_2_ solubility in saline and hypersaline systems, and the ability of extracellular NaCl levels to influence metabolism. We screened unialgal isolates obtained from hypersaline ecosystems in the southwest United States and identified two distinct halophilic strains of the genus Tetraselmis (GSL1 and QNM1) that exhibit both robust fermentative and photo H_2_-production activities. The influence of salinity (3.5%, 5.5% and 7.0% w/v NaCl) on H_2_ production was examined during anoxic acclimation, with the greatest in vivo H_2_-production rates observed at 7.0% NaCl. These Tetraselmis strains maintain robust hydrogenase activity even after 24 h of anoxic acclimation and show increased hydrogenase activity relative to C. reinhardtii after extended anoxia. Transcriptional analysis of Tetraselmis GSL1 enabled sequencing of the cDNA encoding the FeFe-hydrogenase structural enzyme (HYDA) and its maturation proteins (HYDE, HYDEF and HYDG). In contrast to freshwater Chlorophyceae, the halophilic Tetraselmis GSL1 strain likely encodes a single HYDA and two copies of HYDE, one of which is fused to HYDF. Phylogenetic analyses of HYDA and concatenated HYDA, HYDE, HYDF and HYDG in Tetraselmis GSL1 fill existing knowledge gaps in the evolution of algal hydrogenases and indicate that the algal hydrogenases sequenced to date are derived from a common ancestor. This is consistent with recent hypotheses that suggest fermentative metabolism in the majority of eukaryotes is derived from a common base set of enzymes that emerged early in eukaryotic evolution with subsequent losses in some organisms.

## Introduction

The phylogenetically unrelated NiFe- and FeFe-hydrogenases have convergently evolved to catalyze the reversible reduction of protons to H_2_ (2H^+^+2e^−^< = >H_2_) [1]. Several recent studies have documented the diversity of hydrogenase-encoding genes in environments that span a broad range of geochemistry [2]–[8]. In some systems, e.g., terrestrial or marine hydrothermal communities, H_2_ oxidation has been suggested to represent the primary mechanism of energy conservation [9], [10]. Yet, in other systems, e.g., terrestrial or intertidal phototrophic communities, H_2_ evolution appears to be of critical importance to the functioning of the assemblage, in particular at night when the systems become net sources of H_2_ [11]–[13].

Biological H_2_ production requires low-potential reducing equivalents derived from either fermentative pathways that oxidize fixed carbon (typically carbohydrates), or from photosynthetic pathways [14]–[19]. Several eukaryotic algae generate fermentative H_2_ during dark, anoxic acclimation as part of a suite of fermentative pathways that catabolize carbohydrates to alcohols, organic acids and H_2_, which are secreted. These metabolites likely provide a rich source of carbon building blocks and reducing equivalents to organisms inhabiting ecological niches adjacent to the algae, which are responsible for the majority of primary productivity during the day. Algae are also able to use reducing equivalents from the photosynthetic electron transport chain under some conditions to directly reduce hydrogenases at the level of ferredoxin without the input of ATP, a pathway that is theoretically regarded as the most efficient biological means to transform the energy in sunlight to H_2_ for biotechnological applications [19]–[23]. To date, only FeFe-hydrogenases have been unambiguously identified in algae, with organisms such as Chlamydomonas reinhardtii encoding truncated enzymes with only the catalytic H-cluster; a 4Fe4S cluster linked via a bridging cysteine to a two Fe center coordinated by CN^−^/CO ligands and a bridging dithiolate. In contrast, Chlorella variabilis NC64A encodes two hydrogenase enzymes with both the H-cluster catalytic domain and an F-cluster domain that coordinates additional FeS clusters that putatively function in electron transfer [18], [19], [24]–[26].

Despite widespread interest in algal H_2_ production, contemporary research is focused almost exclusively on freshwater species of Chlamydomonas, Scenedesmus, and Chlorella, with C. reinhardtii being the model system for the vast majority of algal hydrogenase research [27], [28]. Significant advances in H_2_ photoproduction from C. reinhardtii have recently been reported [29]–[33]; whereas relatively few studies have examined H_2_ production from marine or halophilic algae [34]–[36].

Halophilic algae offer several advantages for large-scale algal H_2_ production. First, the use of halophilic algae will enable H_2_ production in readily available salt water, thereby minimizing the potential use of limited fresh water resources. Secondly, gas solubility is reduced in aquatic saline systems [37], which is potentially advantageous because the levels of soluble O_2_, a potent inhibitor of most FeFe-hydrogenases [38], are diminished in salt water relative to fresh water. Hydrogen is also less soluble in saline systems and therefore more easily removed. Lastly, ATP hydrolysis is increased in saline media to maintain proper cellular ion gradients. Hydrogenase activity is an ATP neutral process, and photosynthetic electron transport has been shown to decline as photosynthetic ATP levels rise during H_2_ photoproduction in some freshwater algae [39], [23]. Salt stress represents a potential mechanism to alleviate ATP accumulation, which can inhibit the metabolic pathways supplying reductant to hydrogenase.

To further expand our understanding of H_2_ metabolism in extreme environments, and to potentially discover novel organisms with enzymes that have superior biotechnological attributes, we initiated an effort to isolate hydrogen-producing organisms from hypersaline ecosystems. Although salt water systems are ubiquitous around the world, relatively little is known regarding H_2_ metabolism in saline systems, or whether unique enzyme features are necessary for activity in halophiles [40], [41].

## Methods

### Algal strains and growth conditions

Halophilic algae from the genus Tetraselmis (Chlorophyceae) were isolated from water samples collected at (a) a roadside pool bordering the Great Salt Lake (GSL), Utah, USA, with a salinity of 6.0% w/v (Tetraselmis GSL1) and (b) a hypersaline pond with a conductivity reading of 660 mS/cm near Quemado, New Mexico, USA [42] (Tetraselmis QNM1). GSL samples were taken in conjunction with the Great Salt Lake Institute, which holds a permit for sampling on Utah state lands, granted by the Utah Department of Natural Resources, Division of Forestry, Fire and State Lands. Water sampling at the New Mexico site was conducted on public lands administered by the Bureau of Land Management, and did not require a permit nor involve endangered or protected species. Both Tetraselmis isolates have been submitted to the University of Texas (UTEX) algal culture collection.

Isolates were routinely cultured in f/2 medium amended with Booth Bay sea water (National Center for Marine Algae and Microbiota, NCMA), at pH 8.0 [43] and 3.5% of salinity. Where indicated, salinities were increased to 5.5% or 7.0% w/v with NaCl addition. Cultures were maintained at 29°C, without agitation at a constant illumination of ∼30 µmol m^−2^ s^−1^ of photosynthetically active radiation (PAR) by cool-white fluorescent lights. Cell counts were assessed using a Z2 Coulter cell and particle counter (Beckman-Coulter).

C. reinhardtii strain CC124 (nit^−^, mt^−^) was obtained from the Chlamydomonas Genetic Center (http://www.chlamy.org/) and grown in tris–acetate–phosphate (TAP) medium pH 7.2 [44], and shaken at 120 rpm under constant fluorescent irradiance (80 µmol m^−2^ s^−1^ of PAR by GE Ecolux 6500K T5 bulbs). Cells were harvested during mid-logarithmic growth (16–20 µg Chl/ml) for measuring in vitro hydrogenase activities and fermentative H_2_ production.

### Chlorophyll and total protein determination

Total chlorophyll was determined spectrophotometrically by extraction in 100% methanol for the Tetraselmis strains and 95% ethanol for C. reinhardtii [44]. Tetraselmis cells were washed with 2 volumes of MilliQ water to remove salt prior to pigment extraction. Total chlorophyll was selected as the standard in normalizing hydrogenase activity with the light-absorbing capacity of isolates.

Total protein content was analyzed using a Modified Lowry Protein Assay (Pierce) according to the manufacture's instructions. Cell pellets were washed with 2 volumes of MilliQ water and then solubilized in 0.5% SDS. Total protein was quantified using BSA solubilized in 0.5% SDS to generate a standard curve.

### Anaerobic induction

For H_2_-photoproduction measurements, 75 ml of mid-log phase cell cultures were grown at the indicated NaCl levels (3.5%, 5.5% and 7.0% w/v), concentrated by centrifugation at 3716 × g for 15 min at 25°C, resuspended in 1 ml of f/2 medium (at the same salinity used for culturing) and transferred to 16-ml glass serum vials covered with aluminum foil to exclude light. For fermentative metabolite analyses, dark H_2_-production and CO_2_-evolution measurements, 50 ml of mid-log phase cell cultures were harvested, resuspended in 1 ml of fresh medium, and transferred to 16-ml glass serum vials covered with aluminum foil. For in vitro hydrogenase activity assays (see below), 1 ml of mid-log liquid cell cultures was directly transferred to anaerobic vials. For C. reinhardtii, in vitro hydrogenase activity and fermentative H_2_ production, 5 ml of cells from mid-log cultures were harvested by centrifugation (3190 × g for 10 min) and resuspended in 1 ml of anaerobic induction buffer (AIB; 50 mM potassium phosphate buffer, pH 7.2, 3 mM MgCl_2_), then transferred to 13-ml glass serum vials covered with aluminum foil. All vials were sealed with septa, purged for 30 min with ultra-high purity argon and kept sealed in the dark for 4 h or 24 h, as indicated.

### H_2_-photoproduction measurements

Maximum in vivo H_2_-photoproduction rates were determined using a custom-built Ag/AgCl polarographic electrode system (ALGI). H_2_- and O_2_-photoproduction rates were measured simultaneously with two YSI 5331A electrodes (YSI Incorporated, Yellow Springs, OH, USA), poised at +/− 0.6 V, in a water-jacketed (25°C) assay chamber. O_2_ and H_2_ electrodes were calibrated between each measurement using f/2 medium (at the respective salinity of tested cells) saturated by atmospheric O_2_ or by ultra-high purity 5.3% H_2_ (Ar balance), respectively. Pure Ar purging was used to determine electrode baselines and to sparge the assay chamber containing 0.8 ml of f/2 medium of O_2_. 0.2 ml of 4 h and 24 h anaerobically induced cells were then introduced into deoxygenated buffer in the sample chamber. After a 30 s dark acclimation, cells were illuminated for 30 s, using saturating LED (Luxeon III Star, Lumileds) irradiance of 2000 µmol photons m^−2^ s^−1^. H_2_-photoproduction rates were calculated from the initial slope during the first 10 s of illumination from the H_2_-dependent current increase.

### In vitro hydrogenase activity

In vitro Tetraselmis GSL1 and QNM1 H_2_-production activity assays were performed by transferring 1 ml of 2X methyl viologen (MV) buffered solution (10 mM MV, 50 mM potassium phosphate, pH 6.9, and 0.2% triton X-100) and 0.2 ml of reduced sodium dithionite solution (100 mM in 30 mM NaOH) to 1 ml of cells grown in f/2 at the indicated salinities and acclimated to anoxic conditions in f/2 medium at the same salt levels used for culturing for 4 h or 24 h in sealed, Ar-purged 16-ml vials. For the data in Table S1, the in vitro hydrogenase activity protocol was modified for direct comparison of Tetraselmis in vitro hydrogenase activity to the in vitro hydrogenase activity from the fresh water alga C. reinhardtii using final MV assay solutions that were equivalent in salt concentrations for the different species. To obtain the same final assay buffer composition, C. reinhardtii cells grown in TAP medium, were resuspended in 1 ml of AIB and acclimated to anoxic conditions for 4 or 24 h in sealed, Ar-purged 13-ml vials. 1 ml of either a 2X MV solution (10 mM MV, 50 mM potassium phosphate, pH 6.9, and 0.2% triton X-100), used as a control, or 2X MV-f/2 adjusted solutions (10 mM MV, 50 mM potassium phosphate, pH 6.9, and 0.2% triton X-100 dissolved in f/2 medium and adjusted to 3.5%, 5.5% or 7.0% NaCl) was added to measure hydrogenase activity. To 1 ml of the Tetraselmis cells grown and anaerobically induced in f/2 medium at 3.5%, 5.5% or 7.0% NaCl, 1 ml of 2X MV-AIB (10 mM MV, 100 mM potassium phosphate, pH 6.9, 3 mM MgCl_2_) was added. 0.2 ml of reduced sodium dithionite (100 mM in 30 mM NaOH) was then added to initiate hydrogenase mediated H_2_ production in all assays. The reactions were incubated (37°C) in a shaking water bath and H_2_ evolution measured for C. reinhardtii, and the Tetraselmis strains by gas chromatography (GC) using a Hewlett Packard Series II 5890 instrument fitted with a Restek 5 Å Molecular Sieve 80/100 6′ 1/8″ column and a thermal conductivity detector with Ar as the carrier gas. The resulting signal was integrated using ChemStation software and H_2_ was quantified using a standard curve generated from known quantities of H_2_.

### Dark fermentative H_2_, CO_2_ production

Following acclimation of cells to dark, anoxic conditions for 4 or 24 h, H_2_ production was measured by withdrawing 0.2 ml of headspace gas and analyzing by GC for both C. reinhardtii and the Tetraselmis strains. CO_2_ production was also measured from cells acclimated to dark, anoxic conditions (0.5, 4 and 24 h). To sealed vials containing acclimated cells, 1 ml of 1 M HCl was added. The acidified cell suspension was then shaken vigorously to liberate CO_2_ into the vial headspace, and CO_2_ (0.2 ml injection) was quantified by GC (Hewlett Packard 5890 series II) using a Supelco column (80/100 PORAPAK) coupled to a thermal conductivity detector using He as the carrier gas. The resulting signal was integrated using ClassVP software, and the gas was quantified using a standard curve generated from known quantities of CO_2_.

### Dark fermentative metabolite analyses and intracellular sugar content

After 4 or 24 h of dark-anoxic acclimation, fermentative products were analyzed by high pressure liquid chromatography (HPLC). Samples were collected after centrifugation of acclimated cells (3 min at 17000 × g) and the resulting supernatant filtered through a silicon filter (0.45 µm). Filtrate (25 µl) was injected (thermostated sample tray held at 10°C) onto an HPLC column (fermentation monitoring column (BioRad), 150×7.8 mm, 8 mM H_2_SO_4_ as eluent, flow rate of 0.5 ml/min, column operating temperature of 45°C, refractive index (RI) detector operating temperature 50°C, in parallel with a photodiode array detector (PAD) at 210 nm). Resulting signals were integrated and metabolites quantified using a standard curve generated from standards for each metabolite detected. The remaining cell pellet was used for determining cellular dry weights and the reducing carbohydrate content. Cell pellets were resuspended in the same volume of MilliQ water, with 100 µl used for total quantification by the anthrone assay as described previously [25], and the remaining cells used for dry weight determination.

### Genomic DNA, total RNA isolations and sequencing

Genomic DNA from Tetraselmis GSL1 and Tetraselmis QNM1 was isolated from 15 ml of cell culture using a phenol:chloroform extraction protocol as described previously [45]. Total RNA was isolated from 20 ml of 100-fold concentrated mid-log Tetraselmis GSL1 cells grown in f/2 at 3.5% salinity and anaerobically induced in 60 ml sealed, Ar-purged vials for 4 h. Fermentative H_2_ production was confirmed by GC before RNA isolation. Briefly, a cell pellet was obtained by centrifugation (3716 × g for 10 min at 25°C), washed with 2 volumes of MilliQ water followed by centrifugation, then resuspended in 5 ml of Plant RNA Reagent (Invitrogen) with RNA isolated according to the manufacturer's instructions for small scale RNA isolation, with the exception that all reagent volumes were multiplied by a factor of 10. Total RNA was concentrated using the RNeasy MinElute Cleanup Kit (Qiagen), according to the manufacturer's instructions. RNA quality was confirmed via 2% agarose gel electrophoresis and quantified using a NanoDrop® ND-1000 (Thermo scientific), as well as by fluorescence using Quant-iT™ RiboGreen® RNA Reagent and Kit (Invitrogen). All purification steps were done with RNase free reagents.

Total RNA was submitted for sequencing to the National Center for Genome Resources (NCGR, Santa Fe, NM) as part of the Marine Microbial Eukaryote Transcriptome Project (Gordon and Betty Moore Foundation). RNA libraries were prepared from total RNA isolated using the standard Tru-Seq™ RNA protocol - poly-A+ selection (Illumina) with an insert size of ∼200 bp and sequenced from both ends (paired-end reads 2×50-nt) on the Illumina Hi-Seq 2000. The total sequence data generated for each sample (MMETSP0419_1 & 2) was approximately 2.5 Gbp. Reads were filtered for quality (Q15) and read length (0.5).

### Transcriptome Assembly

AbySS [46] was used to generate 20 assemblies across a k-mer sweep from 26 to 50 nt. These assemblies were then subjected to gap closing with GapCloser v 1.10 (Short Oligonucleotide Analysis Package, SOAPdenovo [47]). Gap closed assemblies from the k-mer sweep were reconciled into one assembly using miraEST [48].

### Identification of hydrogenase structural and maturation genes

Hydrogenase structural and maturation genes within the Tetraselmis GSL1 transcriptome assembly were identified using tBLASTn (NCBI). HYDA, HYDEF and HYDG from Chlamydomonas reinhardtii (CAC83731, AAS92601, AAS92602, respectively) and Chlorella variabilis NC64A sequences (AEA34989, EFN57384, EFN57653, respectively) were used as search queries. Assemblies of all identified homologs were manually inspected. RNAseq reads were mapped back to these assemblies using GSNAP (Genomic Short-read Nucleotide Alignment Program) to determine coverage and transcript variation. Mapped reads were reassembled with Geneious (v.5.5.7, Biomatters) to validate the AbySS assembly.

To verify the presence of an unfused HYDE gene, PCR amplification of genomic Tetraselmis GSL1 DNA was used to amplify a region of the unfused, independent HYDE from the 3′-coding region (EU-F1 5′CGACAAGAAGGCCCACCTGGAGA3′) to the 3′UTR of HYDE (EU-R1 5′ GCGTACCTCGCCTGCCCTTACTA 3′), which spans the unfused HYDE stop codon. To verify the presence of the HYDEF fusion in gDNA, primers corresponding to the 3′-region of the HYDE segment in the HYDEF fusion (EF-F1 5′ GTCCCGCTACCTTGTCCGCATTG 3′) and to the 5′-region of HYDF in HYDEF (EF-R2 5′GAATGTGTGCCGAGCTGTGCT 3′) were used, which spans the HYDEF fusion sequence and does not contain a stop codon after the HYDE gene segment. PCR conditions included: polymerase activation: 95°C 2 min, 40 cycles: denaturing 95°C 20 s, annealing 60°C 20 s, elongation 72°C 30 s, and a final elongation at 72°C for 5 min using the KOD DNA polymerase and master mix (Promega). Amplified products were sequenced using the Davis DNA sequencing facility (Davis Sequencing). All retrieved sequences have been deposited in GenBank under the accession numbers: KC820787-KC820794, and KC832401.

### Molecular phylogeny

DNA was isolated from algal colonies grown on agar using the UltraClean Soil DNA Isolation Kit (MoBio Laboratories). Partial 18S SSU rRNA genes (>1350 and 1120 bp, respectively), for Tetraselmis GSL1 and Tetraselmis QNM1 were amplified and sequenced from each alga using the eukaryote-specific primers 360FE and 1492RE [49]. Sequences were aligned using the NCBI BLAST algorithm queried against the nr/nt database. The complete Tetraselmis GSL1 18S SSU rRNA gene (1802 bp) was attained from transcriptome data and deposited in GenBank (accession no. KC820794).

The phylogenetic position of HYDA relative to representatives available in databases [50] was determined using maximum likelihood approaches similar to those described previously [7], [51]. Briefly, HYDA homologs were aligned using the Gonnet substitution matrix specifying a gap opening penalty of 13 and a gap extension penalty of 0.05. The alignment block was trimmed to contain only the aligned positions corresponding to the H-cluster only homolog, HYDA1, from C. reinhardtii (CAC83731). The phylogenetic position of HYDA from Tetraselmis GSL1 was determined using PhyML (ver. 3.0) [52] specifying the LG substitution model with gamma distributed rate variation and a proportion of invariable sites, as recommended by ProtTest (ver. 3.2.1) [53]. A HYDA paralog, i.e., Nar1, from Saccharomyces cerevisiae, served as the outgroup in the phylogenetic analysis of HYDA.

To further increase the resolution of our phylogenetic analysis of the FeFe-hydrogenase identified in the transcriptome of Tetraselmis GSL1, we compiled homologs of HYDA, HYDE, HYDF, and HYDG from all eukaryotes with available genomes. All homologs were aligned and trimmed as described above. Alignment blocks were concatenated using base functions within PAUP (ver. 4.0) [54] and the phylogenetic position of the HYDAEFG concatenated alignment was determined using PhyML as described above. HYDAEFG homologs from Bacteroides thetaiotaomicron VPI-5482, served as the outgroup in this phylogenetic analysis.

## Results

### Identification of hydrogenase activity in halophilic strains of Tetraselmis

**Figure 1:**
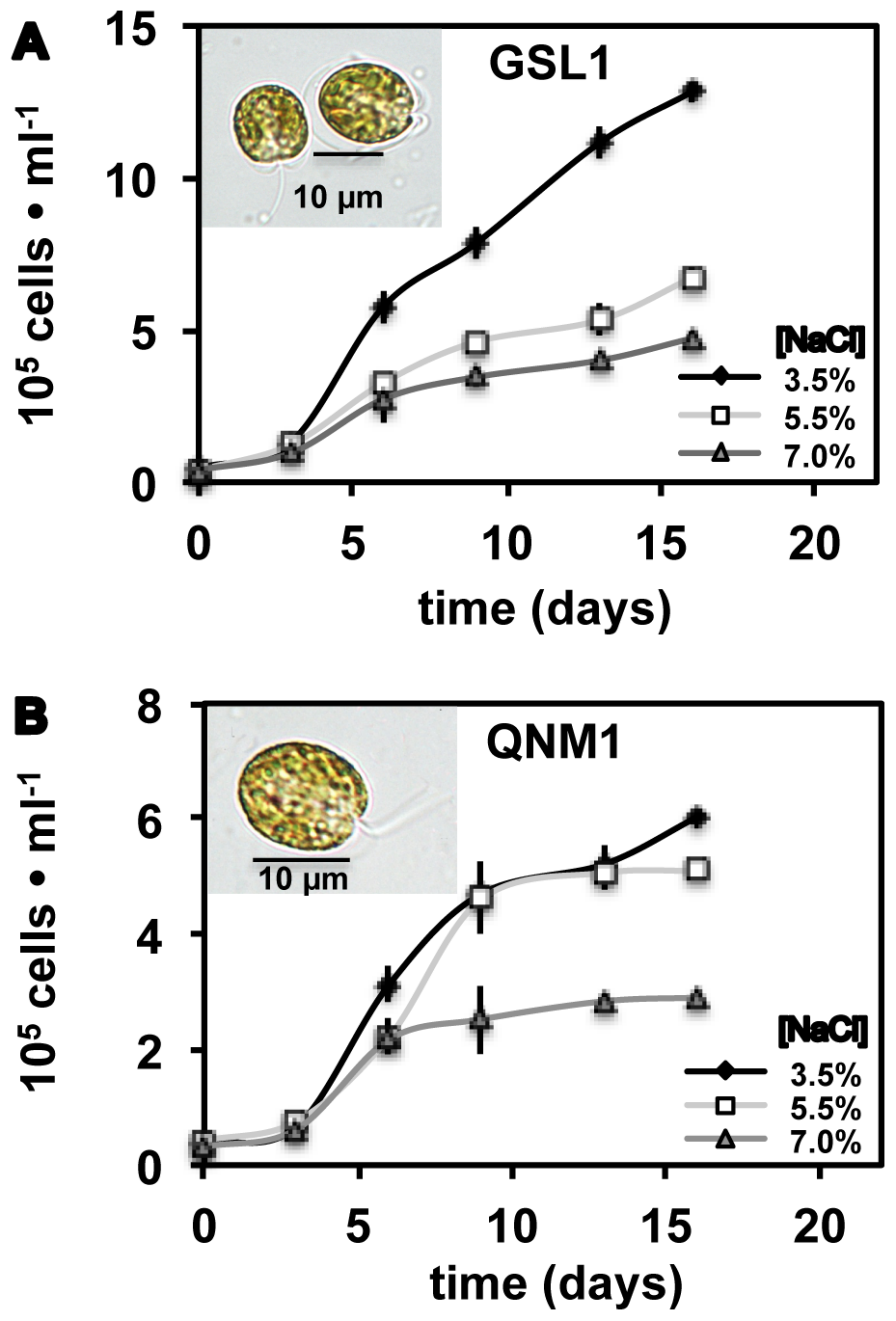
Cell densities in f/2 medium adjusted to 3.5%, 5.5% or 7.0% NaCl (w/v). Tetraselmis GSL1 (A) and Tetraselmis QNM1 (B) cell numbers were determined using a Beckman Z2 Coulter particle counter. Error bars represent standard deviations from 6 experimental repetitions. Representative light microscope images from cells grown at 3.5% NaCl are inset.

To assess whether algae with hydrogenase activity are present in hypersaline environments, water samples were collected from a variety of GSL sites with salinities ranging from 3.5–25%, and unialgal isolates from these water samples were obtained using flow cytometry. Hydrogenase activity screens of over 40 unialgal isolates recovered from GSL using the reduced MV assay revealed that the most robust hydrogenase activity was detected from a tetraflagellate alga that morphology and 18S rRNA gene analysis indicated to be a novel strain of Tetraselmis (Tetraselmis strain GSL1), isolated from a roadside pool bordering GSL with a salinity of 6.0% w/v. Notable hydrogenase activity was not detected in any of the isolated strains of Dunaliella, which have been shown to be the numerically dominant alga in several GSL environments [55]. We also obtained an axenic culture of another halophilic strain of Tetraselmis (Tetraselmis QNM1), which was isolated from a hypersaline pond in New Mexico and was available in the Colorado Center for Biofuels and Biorefining algal culture collection [42]. Tetraselmis GSL1 and Tetraselmis QNM1 18S rRNA genes are most similar to the recently described Tetraselmis indica, isolated from salt pans in Goa, India [56]. Both Tetraselmis GSL1 and Tetraselmis QNM1 remained viable at NaCl concentrations greater than 10%, but growth was reduced at these NaCl levels. Therefore, subsequent characterization of hydrogenase activities in the Tetraselmis isolates were done at a comparable ocean salinity of 3.5%, as well as at the increased salinities of 5.5% and 7.0% (the later concentrations are similar to the salinity where this taxon was isolated) to determine the effects of elevated NaCl concentrations on hydrogenase activity. As shown in Figure 1, the highest growth rates and cell densities are observed in medium with 3.5% NaCl, with growth rates declining as NaCl concentration is increased. In the case of Tetraselmis QNM1, cells reached stationary phase earlier when cultured at 7.0% NaCl, yet final cell densities were approximately 50% lower.

### Hydrogenase activity in vitro as a function of salinity

**Figure 2:**
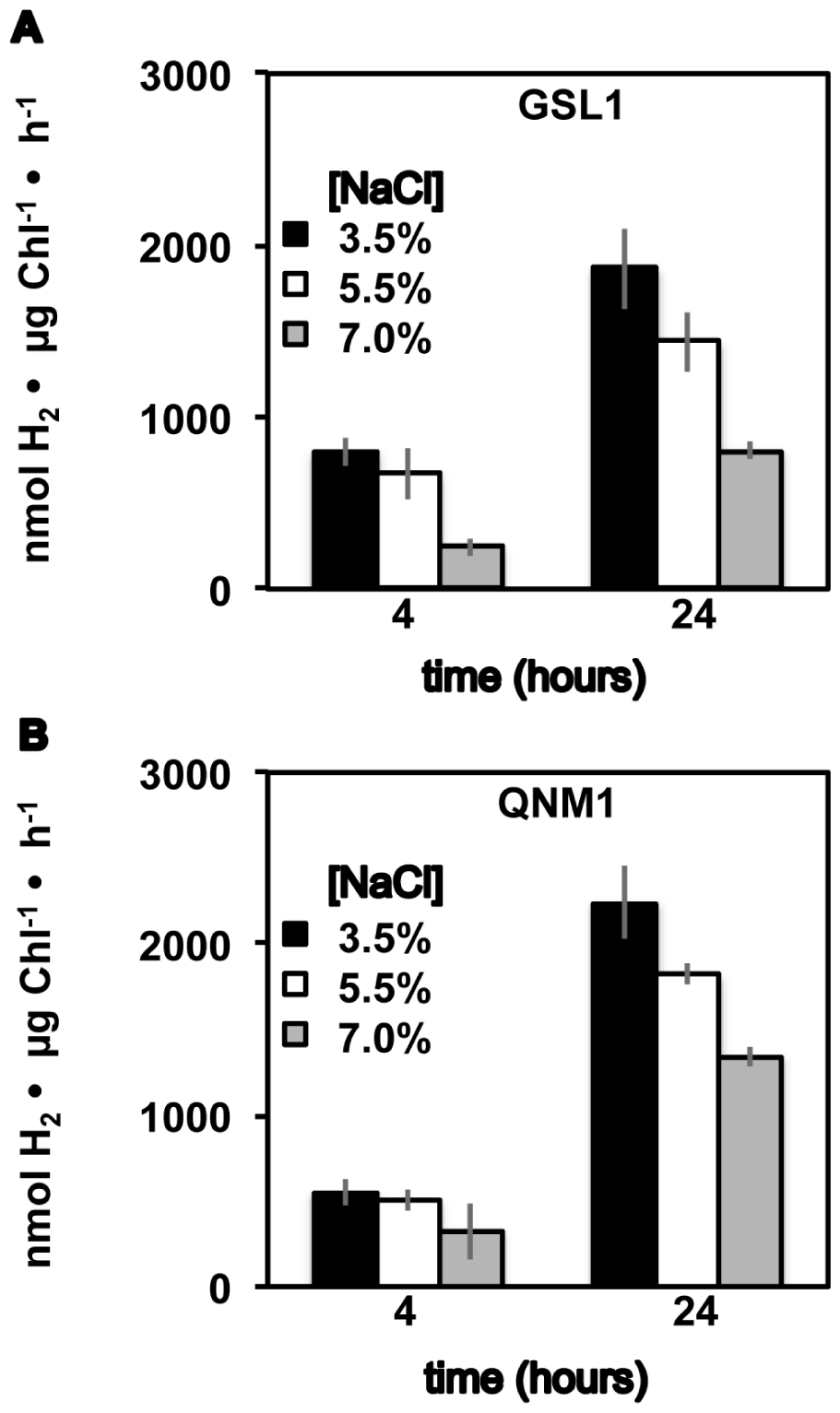
Methyl viologen mediated in vitro hydrogenase activity. Hydrogen production rates from anoxic Tetraselmis GSL1 (A) and Tetraselmis QNM1 (B) cell extracts incubated with reduced dithionite. Cultures were grown and then acclimated to dark, anoxic conditions in f/2 medium at the indicated NaCl levels (w/v). Error bars represent standard deviations from 8 experimental repetitions.

Given previous data indicating that salt levels impact algal metabolism and protein expression [41], [57]–[60], hydrogenase activity was assessed after culturing at 3.5%, 5.5% and 7.0% NaCl concentrations. Cells were acclimated in the dark under anoxic conditions for 4 or 24 h prior to measurement of in vitro hydrogenase activities using cell extracts and reduced MV. As shown in Figure 2, significant hydrogenase activity is observed in cultures grown at all three NaCl concentrations, with a diminution of total in vitro activity as extracellular NaCl levels increase for both strains. The in vitro hydrogenase activity measured at 3.5% NaCl is more than an order of magnitude greater than the in vitro hydrogenase activity typically reported on a chlorophyll basis for C. reinhardtii [61]. As the ionic strength of the assay buffer can influence the measured in vitro hydrogenase activity [62], we tested whether the differences in hydrogenase activity can be attributed to the higher ionic strength used to measure the Tetraselmis MV-mediated hydrogenase activity. As shown in Table S1, when both the Tetraselmis strains and C. reinhardtii are assayed using equivalent final MV assay solutions at variable salt levels, the Tetraselmis strains still show in vitro hydrogenase activity that is about an order of magnitude greater than the rates from C. reinhardtii, with the greatest differences emerging at the 24 h time point (up to ∼30 fold at 24 h and 1.6% NaCl). We also assessed hydrogenase activity as a function of total protein levels (Table S1), and again hydrogenase activity was approximately an order of magnitude greater than is observed from C. reinhardtii after 24 h of anaerobic induction, and ∼5 times higher at 4 h of anaerobic induction, when the NaCl levels in the MV assay are equivalent.

### Hydrogen photoproduction activities in vivo

**Figure 3:**
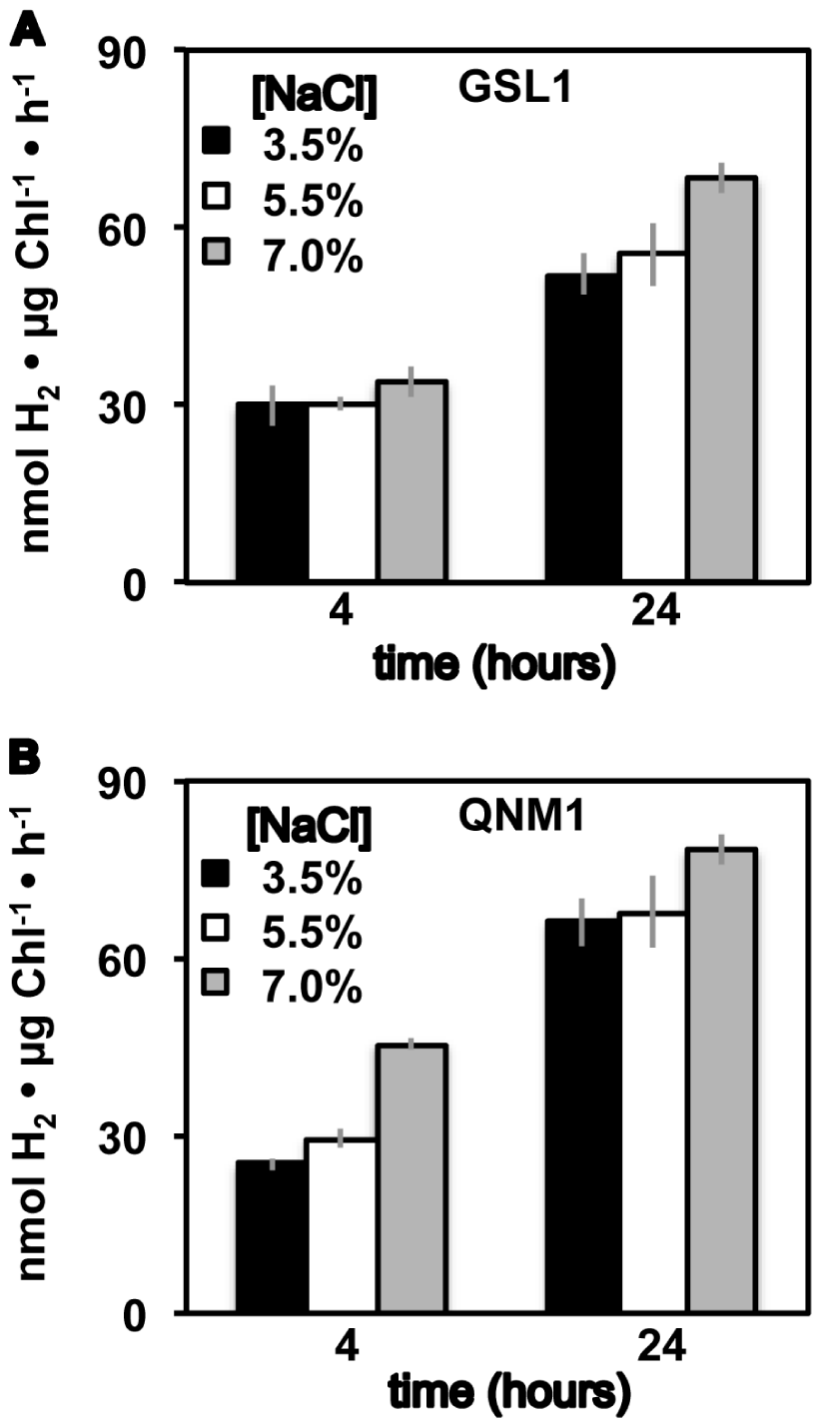
In vivo H_2_-photoproduction rates. Hydrogen production rates after illumination of dark, anoxic cultures of Tetraselmis GSL1 (A) and Tetraselmis QNM1 (B). Bar graphs indicate maximal initial H_2_-photoproduction rates measured using a custom built Clark-type electrode. Cells were grown and acclimated to dark, anoxic conditions in f/2 medium at the indicated NaCl levels (w/v). Error bars represent standard deviations from 10 experimental repetitions.

Hydrogen photoproduction was assayed using a custom-built, Clark-type electrode [61] (Fig. 3). Both Tetraselmis strains showed significant H_2_-photoproduction rates (albeit ∼2-fold less than those attained in C. reinhardtii, when rates were normalized to total chlorophyll at 4 h of anoxic acclimation), indicating that hydrogenase activity is coupled to the photosynthetic electron transport chain, as is observed in several species of the Chlorophyceae [50]. In contrast to the results of the MV in vitro hydrogenase assays, H_2_ photoproduction in Tetraselmis GSL1 increased slightly as a function of salinity. Given that the in vivo H_2_-photoproduction rates are significantly lower (∼30 fold) than the measured in vitro (i.e., MV assays) rates, excess enzyme capacity exists under these conditions relative to the amount of reductant supplied by the photosynthetic electron transport chain for H_2_ production. These results suggest that although the amount of active enzyme decreases as a function of salinity, the efficiency of hydrogenase coupling to the photosynthetic electron transport chain increases. Interestingly, in C. reinhardtii and other green algae, the in vivo hydrogenase activity levels are frequently similar to the in vivo H_2_-photoproduction rates [61], which is not observed in the two Tetraselmis isolates studied. Similar to the in vitro assays, H_2_-photoproduction rates increased (∼2–3 fold) at 24 h relative to 4 h of dark, anoxic acclimation. H_2_-photoproduction rates were higher (∼3–4 fold) than those of C. reinhardtii (Table S1) when normalized to total chlorophyll following 24 h of dark, anoxic acclimation [61].

### Fermentative metabolite analysis

**Figure 4:**
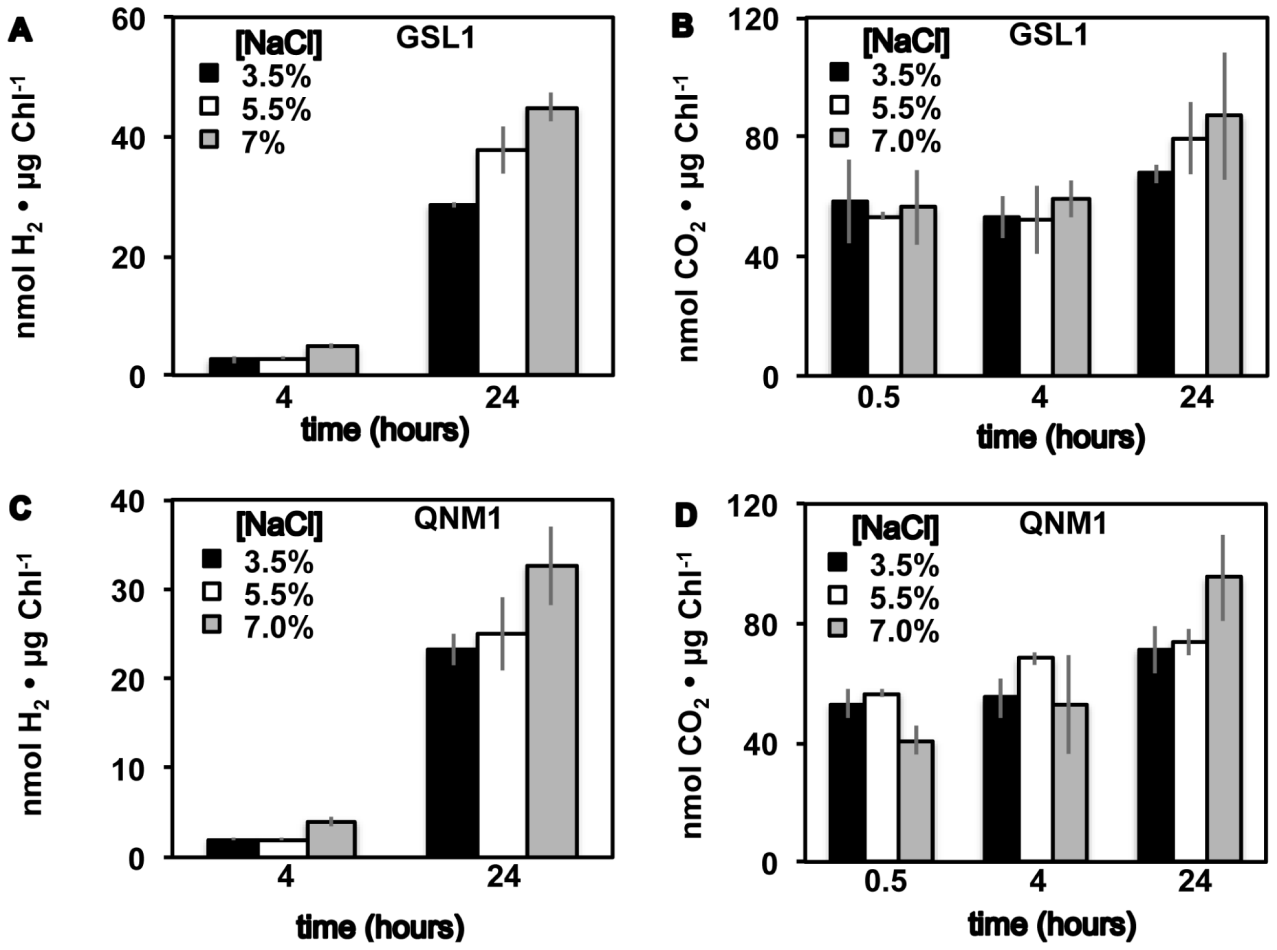
H_2_ and CO_2_ levels after dark, anoxic acclimation. H_2_ (A, C) and CO_2_ (B, D) detected from sealed vials during dark, anoxic acclimation of Tetraselmis GSL1 (A, B) and Tetraselmis QNM1 (C, D). Cells were grown and acclimated to anoxia in f/2 medium adjusted to the indicated NaCl levels (w/v). Error bars represent standard deviations from 14 (H_2_) and 6 (CO_2_) experimental repetitions.

**Figure 5:**
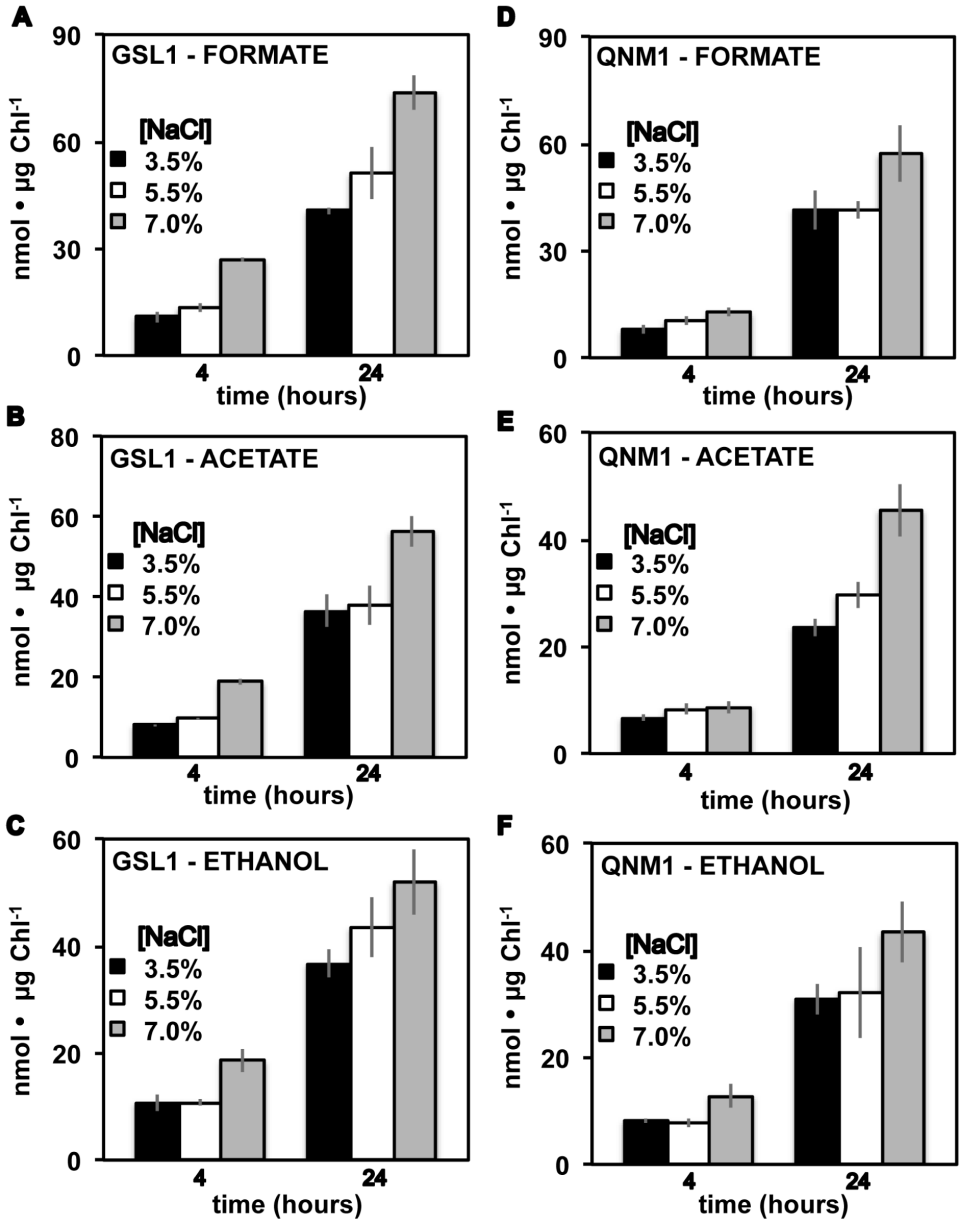
Accumulation of selected metabolites in extracellular medium. Bar graphs indicate the levels of secreted formate, acetate and ethanol from cultures of Tetraselmis GSL1 (A, B, C) and Tetraselmis QNM1 (D, E, F), respectively. Cells were grown and acclimated to dark, anoxic conditions in f/2 medium adjusted to the indicated NaCl levels (w/v). Error bars represent standard deviations from 8 experimental repetitions.

Fermentative H_2_ was detected in the gas phase of cultures of both Tetraselmis GSL1 and Tetraselmis QNM1 following dark, anoxic acclimation (Fig. 4), with H_2_ yields being among the highest reported to date for green algae (e.g., ∼20 fold higher on a per chlorophyll basis than those observed in C. reinhardtii (Table S1)). Both Tetraselmis strains also accumulated CO_2_ in the headspace (Fig. 4) during fermentation, as would be expected if H_2_ production was linked to the oxidative decarboxylation of pyruvate by pyruvate-ferredoxin oxidoreducase with subsequent electron transfer from ferredoxin to hydrogenase [63], [64]. The identification of extracellular formate, acetate, and ethanol in the medium following acclimation to dark, anoxic conditions (Fig. 5) suggests that these algae employ a classic heterofermentation pathway, similar to that which has been observed in other green algae [18]. Metabolite analyses indicate up to a 4-fold increase in fermentative product accumulation at 24 h of dark, fermentation relative to 4 h, demonstrating the ability of these Tetraselmis strains to maintain prolonged anoxic metabolism.

### Carbohydrate metabolism

Total sugar content was determined using the anthrone assay [25] since it is likely that carbohydrates serve as fermentable substrates in Tetraselmis. As shown in Table 1, concentrations of total reducing sugars increase as a function of higher salinity, consistent with the accumulation of organic osmolytes, as has been observed in other halophilic algae and cyanobacteria [41], [65], [66]. During acclimation to dark anoxia, carbohydrate degradation was observed concomitant with fermentative product secretion. Approximately 30–50% of the carbohydrate accumulated was catabolized at the end of 24 h of anoxia. Paralleling the increase in total carbohydrate as a function of salt concentration, an increase in fermentative metabolites is observed at higher NaCl concentrations, indicative of increased metabolic flux through fermentation. Salt-induced osmotic stress has previously been reported to increase carbohydrate and lipid content in green algae and cyanobacteria, and salt-stress cycling has previously been applied for improving biofuel yields [65], [66].

**Table 1:** Total reducing sugars detected using the Anthrone Assay.

strain - salinity	Time	µg tot sugars/µg Chl	SD
GSL1 - 3.5%	0 h	55.11	±10.70
	4 h	50.62	±9.12
	24 h	38.00	±7.45
GSL1 - 5.5%	0 h	63.72	±14.52
	4 h	61.13	±12.07
	24 h	39.50	±3.47
GSL1 - 7.0%	0 h	94.73	±9.25
	4 h	85.70	±13.04
	24 h	49.55	±8.21

strain - salinity	Time	µg tot sugars/µg Chl	SD
QNM1 - 3.5%	0 h	58.66	±4.59
	4 h	57.09	±9.54
	24 h	52.10	±2.95
QNM1 - 5.5%	0 h	64.43	±2.12
	4 h	60.62	±8.71
	24 h	52.85	±4.53
QNM1 - 7.0%	0 h	75.90	±8.04
	4 h	51.99	±11.72
	24 h	39.95	±4.59

Intracellular reducing sugar content in cultures of Tetraselmis GSL1 and Tetraselmis QNM1, grown and acclimated to dark, anoxic conditions at the indicated times and NaCl concentrations (w/v).

### Transcriptome analysis and hydrogenase gene isolation

Attempts to amplify Tetraselmis GSL1 HYDA1 from genomic DNA or transcript preparations using degenerate primers [7] were unsuccessful. We therefore used whole transcriptome sequencing from Tetraselmis GSL1 after 4 h of anoxic acclimation for gene discovery [67]. From the transcriptome dataset, full length transcripts with homology to the hydrogenase structural gene (HYDA [24]), as well as the hydrogenase maturases (HYDE, HYDEF, and HYDG [68], [69]) were attained. Only one FeFe-hydrogenase gene (HYDA) that encodes an H-cluster domain, but that lacks any F-cluster domains, was identified. For all other green algae exhibiting hydrogenase activity, and with available transcriptome/genome data, at least two FeFe-hydrogenases are present, with Chlorophytes (e.g., Chlamydomonas and Scenedesmus) encoding H-cluster only enzymes and Chlorella NC64A (Trebouxiophyceae) encoding two FeFe-hydrogenases with both the H-cluster domain and additional F-cluster binding domains [25]. There are several explanations that may account for the detection of only one HYDA homolog: (a) Tetraselmis GSL1 contains only a single HYDA isoform, which would be the first report of a single HYDA isoform in a Chlorophyceaen alga; (b) other HYDA isoform(s) exist in the Tetraselmis GSL1 genome but are not expressed at a sufficient level for detection using our assay conditions; or (c) other HYDA isoform(s) are present in the Tetraselmis GSL1 genome but are no longer expressed and therefore not detectable by transcriptome sequencing.

Also unexpected in the transcriptome data was the finding that a HYDE homolog not fused to HYDF is observed as an independent transcript. To date, all other sequenced green algae with hydrogenase activity contain only a HYDEF fusion, and this is the first time that a transcript encoding an independent HYDE has been observed in a green alga. Tetraselmis GSL1 also has a second transcript encoding a gene with HYDE homology that is fused with HYDF (HYDEF). The independent HYDE transcript has a 3′ untranslated region (UTR) and translation stop site, whereas in the HYDEF fusion, the HYDE portion of the gene is fused in frame with the HYDF coding sequence (Fig. S1). The HYDE section of the HYDEF fusion lacks both the last 19 nucleotides found in the independent HYDE coding DNA sequence (CDS), as well as the HYDE 3′ UTR. This CDS deletion effectively removes the translation stop site and the last 6 amino acids at the C-terminus of HYDE. An intronic sequence at the 3′ end of the HYDE region in HYDEF allows an in frame fusion with the 5′ end of the HYDF gene (Fig. S1). The presence of a single HYDF independent of the HYDEF fusion is not supported due to the lack of transcriptional evidence for an independent HYDF 5′UTR and translation start site; however, we cannot eliminate the possibility of a genomic copy of HYDF not expressed under our conditions.

The presence of a single HYDE gene, as well as a HYDEF fusion was verified by PCR using genomic DNA. Primers were designed to amplify only the HYDE gene (sequences complimentary to the C-terminus region of HYDE coding sequence and to the HYDE 3′ UTR downstream of the stop codon); and another primer set to amplify only the HYDEF fusion (primers corresponding to the C-term region of the HYDE coding sequence and the N-terminus region of the HYDF coding sequence). We confirmed that (a) an independent HYDE gene (not fused to HYDF) with a discrete stop codon and 3′UTR and (b) a fused HYDEF that has neither the HYDE 3′UTR, nor a stop codon after the HYDE coding sequence are encoded in the Tetraselmis GSL1 genomic DNA. Sequencing of a gDNA PCR product that spans the region between HYDE and HYDF within the HYDEF fusion revealed an additional intron not present in the independent HYDE that directly connects to an exon of the HYDF coding sequence. In sum, these data are consistent with a simple fusion between what was the 5′ UTR of HYDF to the last exon of a copy of HYDE, effectively eliminating the stop codon and 3′ UTR of this HYDE, which generates a new intron connecting HYDE and HYDF coding sequences. As a negative control, primers were designed corresponding to the 3′UTR of HYDE and the N-terminal coding sequence of HYDF, and as expected no PCR product was obtained.

The relative expression of each transcript was determined and averaged for two biological replicates (Table 2). The hydrogenase structural gene (HYDA) had the highest expression levels of all hydrogenase related genes and was the 16^th^ most abundant transcript in the entire transcriptome assembly, suggestive of a key role in the anoxic proteome and consistent with the substantial levels of in vitro hydrogenase activity in this alga. Both HYDE and HYDG are expressed at commensurate levels with ∼10-fold fewer transcripts than HYDA. Surprisingly HYDEF is only expressed at ∼10% of the levels of HYDE and HYDG (100-fold less abundant than HYDA).

**Table 2:** HYD gene transcript lengths, relative expression levels and rank with respect to the cellular transcriptome.

Gene	Number of homologs	Transcript length (nt)	Length of predicted protein (AA)	RPKM^a^	Expression rank in transcriptome assembly^b^
HYDA	1	1,825	461	1461±32	16
HYDE	1	2,038	492	118±1.6	655
HYDEF	1	3,446	997	14±3.0	7,088
HYDG	1	2,033	541	113±0.6	631

a Transcript expression level in reads per kilobase per million mapped reads. Given is the average expression and range for two biological replicates.

b Rank of transcript expression when compared to entire transcriptome assembly, with one being the most expressed transcript out of a total of 31,619 transcripts.

Single nucleotide polymorphisms (SNPs) were found in all of the hydrogenase related transcripts (Table 3 and Table S2). The HYDA transcript had the most SNPs detected at 39, while HYDG had only a single SNP. The majority of SNPs are synonymous nucleotide substitutions that result in no amino acid change (Table 3). Of the 34 SNPs detected in the HYDA CDS, only 6 of these result in amino acid changes, all of which occur at the N-terminus end of the peptide and are located outside of active site regions. Genomic sequencing of the fusion region of HYDEF revealed only four SNPs between the HYDE portion of HYDEF relative to the independent HYDE transcript.

**Table 3:** Single nucleotide polymorphisms (SNPs) in HYD transcripts.

Genes	SNPs in CDS	AA changes	Avg. Allele 1 Frequency^a^	Avg. Allele 2 Frequency^a^
HYDA	34	6	50.6±6.6	47.6±5.9
HYDE	0	0	-	-
HYDE/EF^b^	4	2	90.1±2.8	8.3±1.8
HYDG	1	1	49.7	48.6

a Average frequency, in percentage, of all CDS SNPs of given allele found in sequenced transcriptome. For HYDE/EF Allele 1 represents HYDE and Allele 2 represents HYDEF.

b HYDE/EF indicates the comparison of the HYDE region of the HYDE and HYDEF.

### Phylogeny of hydrogenase and maturase enzymes

**Figure 6:**
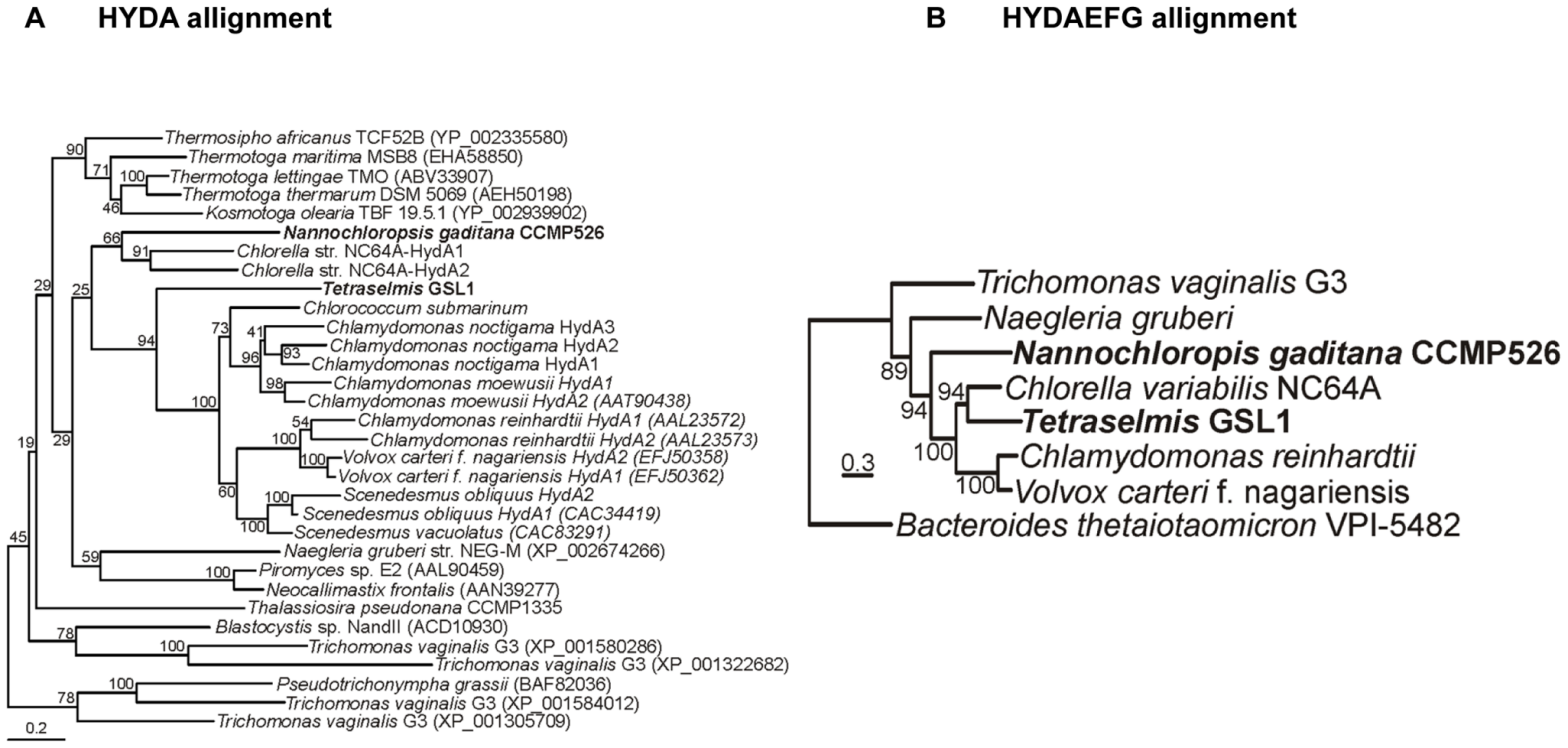
Maximum-likelihood phylogenetic reconstruction of individual HYDA sequences (A) and a concatentation of HYDAEFG (B). Bootstrap support values are indicated at each node. The root in the HYDA (NAR1, paralogs of HYDA) was pruned to conserve space. Given that NAR1 paralogs of HYDEFG do not exist, a different outgroup was chosen (i.e HYDAEFG from Bacteroides thetaiotaomicron) on the basis that the HYDA that it encodes, which branches basal to the eukaryote HYDA lineage.

We recently described the presence of two genes encoding HYDA in the green alga Chlorella variabilis NC64A, which is the only representative of the Chlorophyta known to date that has FeFe-hydrogenases containing F-cluster domains [25]. Additionally, this study determined that all algal hydrogenase H-clusters sequenced to date are monophyletic, indicating a single common ancestor for the incorporation of hydrogenase activity in algal metabolism [25]. We also recently sequenced the genome of the marine alga Nannochloropsis gaditana (Eustigmatophyceae) [70] and observed the presence of a single FeFe-hydrogenase encoding both H- and F-cluster domains in this alga [71]. As shown in Figure 6, rigorous phylogenetic analyses demonstrate that the recently obtained algal HYDA sequences from Tetraselmis GSL1 and N. gaditana are also monophyletic with the other algal FeFe-hydrogenases. This is somewhat surprising given the evolutionary distance between Nannochloropsis and Tetraselmis, and indicates a relatively early origin of algal FeFe-hydrogenases, algal H_2_ metabolism, and possibly algal fermentation pathways. Additionally, as the Nannochloropsis and Tetraselmis hydrogenases are the first full-length marine/halophilic sequences available, this data indicates that hydrogenases in both salt-water and fresh-water algae are derived from a common ancestor.

As expected, HYDA from Tetraselmis GSL1 nested with the Chlorophyceae, supporting the highest max score and smallest e-value obtained when aligned with C. reinhardtii (392 and 9e-136, respectively), compared to C. variabilis NC64A enzymes (367 and 3e-123) or using blastx CDS alignment (best four results Chlamydomonas moewusii, Volvox carteri, Acutodesmus obliquus, all in the Chlorophyceae). Curiously, while HYDA phylogenetic analyses placed Tetraselmis GSL1 as a sister clade of the Chlorophyceae and placed C. variabilis NC64A basal along with the more ancestral N. gaditana, concatemeric analysis of HYDAEFG sequences suggests a sister clade position for Tetraselmis and Chlorella members; N. gaditana branches basal among algae with genome sequences available. The discrepancy associated with the branching order of Tetraselmis GSL1 and N. gaditana HYD phylogeny when either HYDA is analyzed alone or when HYDAEFG are considered together may arise from differences in the number of available sequences used to construct the trees, leading to bias in the concatenated analysis. Alternatively, these results may suggest slightly different evolutionary histories or selective pressures that have acted on the structural enzyme, HYDA, and the maturases, HYDE, HYDF, HYDG.

Previously, it was suggested that the multiple HYDA isoforms present in algal genomes are the result of numerous, independent duplication events within each individual lineage (i.e., multiple HYDA isoforms is not an ancestral trait) [25]. The genome of N. gaditana and the transcriptome of Tetraselmis GSL1 suggest that each of these strains encode a single copy of HYDA. When coupled with HYDA phylogenetic data, which indicates that N. gaditana branches at the base of the algal lineages, these results support the notion that the progenitor of H_2_ metabolism in algae encoded for only a single HYDA isoform. The observation that HYDA from Tetraselmis GSL1 is nested among algae with multiple HYDA isoforms supports the previous claim that independent duplication events led to the multiple isoforms present in these other strains.

## Discussion

Salt water systems have a number of potentially intriguing applications in biological H_2_ production using phototrophic microorganisms [72]. Saline water is readily available and salt concentrations can likely be leveraged to influence metabolism. Because little previous research has examined H_2_ production in marine or halophilic algae, we undertook an effort aimed at identifying and characterizing hydrogenase activity in halophilic algae, and identified two distinct strains of Tetraselmis with fermentative metabolisms and promising H_2_-photoproduction activities. To date, these strains of Tetraselmis are the most robust H_2_-producing phototrophs isolated from high-salt environments; however it should be noted that both are relatively slow growing. Marine phototrophs are responsible for a significant portion of global primary productivity, can be cultured in abundant salt water resources, and diverse species of Tetraselmis are present in several saline ecosystems [73], [56]. Moreover, species of Tetraselmis have emerged as biotechnologically relevant organisms for the production of biofuels such as lipids, ethanol, or starch and for the extraction of higher value biocommodities including vitamins and ceramides [74]–[80], and now H_2_.

We confirmed that the halotolerance of Tetraselmis allows its growth in a wide range of salinities. The ability to grow at ocean salinity (3.5%), as well as more saline environments makes these organisms particularly versatile for biotechnological applications. Interestingly, we observed increases in both fermentative and photo- H_2_ production as salinity increased, despite decreases in in vitro hydrogenase activities. Further studies are necessary to uncover the mechanistic underpinnings of the increase in H_2_ production; however, it is plausible that the higher salt concentrations increase ATP demand, allowing for increased photosynthetic activity in the case of H_2_ photoproduction, or increased fermentative activity in the case of dark, anoxic H_2_ production. Both of these pathways increase ATP synthesis/utilization via pathways that require anoxic terminal electron acceptors, which may explain the increases in H_2_ production. Although further research is necessary to verify or refute this hypothesis, it is clear that salt can be used to increase H_2_ production in these Tetraselmis strains.

Five unique features of anoxic metabolism that have not been observed in more established freshwater, model systems were observed in these Tetraselmis isolates. First, hydrogenase activities accumulated to levels that are an order of magnitude greater than is typically observed in C. reinhardtii and other green algae when assayed on a per unit chlorophyll basis. This level of activity greatly exceeds the reducing equivalents delivered to the enzyme by either photosynthetic or fermentative pathways; suggesting that gains in H_2_ production can be achieved by increasing reductant to the hydrogenase without having to overexpress the enzyme. Second, robust H_2_ production coupled to either the photosynthetic electron transport chain or fermentative pathways can be realized at NaCl concentrations almost two times that of sea water; enabling consideration of salt water resources in efforts to efficiently produce biological H_2_ from algae. Third, although increasing salt levels attenuate in vitro enzyme activity, increases in both H_2_-photoproduction and dark, fermentative H_2_ production are observed as a function of increasing NaCl levels. This demonstrates that salt levels can positively influence H_2_ production in algae. Fourth, hydrogenase activity is robust even after 24 h of dark anoxia, which is in contrast to the reduced hydrogenase activities reported for C. reinhardtii at this point in anoxic acclimation [61]. Carbohydrate analysis indicated that well over half of the available reducing sugars remain within the cells at this point of anoxic acclimation, and it may be that these Tetraselmis strains are “hardwired” to withstand extended periods of anoxia. Fifth, fermentative H_2_ production in the dark is almost 20-fold greater on a per unit chlorophyll basis than reported for C. reinhardtii, suggesting that hydrogenase may be more effectively integrated into fermentative pathways.

In addition to the biotechnological features described, we are also interested in (a) characterizing phototroph metabolism in the dark when anoxia occurs in aquatic “dead-zones” and phototrophs rely on fermentation for ATP production and (b) developing a more informed understanding of the evolution of FeFe-hydrogenases in eukaryotic phototrophs to better define why hydrogenase activity is observed in these oxygenic photosynthetic organisms. Eukaryotic phototrophs with hydrogenase activity typically exhibit anoxic heterofermentation activity in the dark, resulting in the secretion of alcohols, organic acids and H_2_ [18]. These energy carriers and organic building blocks secreted by the algae are likely used by the consortia of heterotrophic microorganisms that coexists with aquatic phototrophs. These metabolites likely influence the population dynamics of the aquatic microbiota over temporal (diurnal, seasonal cycles) and spatial (vertical depth) gradients. Such features warrant further investigation of phototroph fermentation in order to develop an understanding of the environmental cues influencing metabolism, catalog the products secreted by microbial primary producers, and characterize the effects of fermentative product secretion on microbial population dynamics.

Similar to C. reinhardtii, formate, acetate, and ethanol were secreted into culture media and H_2_ and CO_2_ were evolved following dark, anoxic acclimation. Other H_2_ producing algae can secrete different products such as lactate or glycerol after anoxic acclimation [50]. Although different organic acids and alcohols can be secreted, all H_2_ producing algae characterized to date exhibit a form of heterofermentation, which is also observed in the species of Tetraselmis described. Müller et al. recently proposed that a base set of anoxic metabolisms was likely present in the single common eukaryote ancestor, and that most contemporary eukaryotic anoxic metabolisms exhibit at least a subset of these original pathways [81]. Many eukaryotes retain features of these core enzymes, with hallmarks including enzymes such as the FeFe-hydrogenase, pyruvate ferredoxin oxidoreductase, pyruvate formate lyase, lactate dehydrogenase and alcohol dehydrogenase, among other fermentative enzymes enabling the production and secretion of H_2_, organic acids and alcohols [82]. Despite greater than a billion years of evolution and ample opportunity to acquire different modes of anoxic metabolism by lateral gene transfer from bacteria and archaea, most eukaryotes with active anoxic metabolisms leverage either fermentation or relatively simple anoxic respiration pathways. This hypothesis is consistent with our observation that Tetraselmis readily activates heterofermentation pathways during anoxia using a subset of the ∼50 enzymes posited by Muller et al. to be present in the eukaryote common ancestor [81]. The Tetraselmis GSL1 transcriptome (see below) was attained as part of an effort to obtain transcriptomes from hundreds of eukaryotic algae to mine this information for unique gene content in marine algae. When completed, this project will provide a powerful dataset for use in determining whether evidence exists that eukaryotic phototrophs have acquired other anoxic metabolisms outside of the core proposed by Muller et al. by lateral gene transfer, which does not appear to be a frequent occurrence.

Phylogenetic analysis of algal hydrogenases sequenced to date show that these enzymes are all monophyletic, indicative of a common ancestor and that distinct lateral gene transfer of hydrogenase among the various algal lineages has not occurred. To determine whether this is also the case for Tetraselmis hydrogenases and to determine if halophiles acquired distinct hydrogenase enzymes from sources that are unrelated to freshwater algae, we performed in depth transcriptome analyses (n = 2) after 4 h of anoxic acclimation using Tetraselmis GSL1. Two surprising features emerged from these analyses that have not previously been described. First, the anoxic transcriptome of Tetraselmis GSL1 suggests a single FeFe-hydrogenase with only the H-cluster binding domain. Without whole genome sequencing, it is impossible to rule out the possibility that other HYDA homologs are present in the Tetraselmis GSL1 genome. However, if other HYDA homologs are present, their expression is sufficiently low after 4 h of anoxia that we could not detect transcripts in our dataset. This is not consistent with other green algae, such as Chlamydomonas, in which both HYDA1 and HYDA2 transcripts increase after exposure to anoxia and have been detected 4 h after anaerobic induction [63]. The depth of coverage obtained by transcriptome sequencing was high enough to capture HYDA precursor mRNA that contained unspliced introns (data not shown), so it seems unlikely that an alternative HYDA homolog is being expressed at substantive levels and not being detected. It seems likely that Tetraselmis encodes only a single FeFe-hydrogenase, and if so, it is the first green alga described that lacks a secondary duplication of this gene. As several SNPs were found in Tetraselmis GSL1 HYDA, we analyzed the SNP frequency. Our results indicate these 34 nucleotide differences are a result of multiploidy, were localized to the putative transit peptide, and resulted in only 6 AA changes (98.7% identical AA). Chlamydomonas reinhardtii is haploid and the HYDA2 isoform in this alga shares only 68% AA identity to HYDA1 [24]. Moreover, the promoter regions of HYDA1 and HYDA2 are unique and lack significant regions of sequence homology, which may suggest differences in the regulation of gene expression, which has been experimentally verified [83]. This phenomenon is not likely to be the case for the two Tetraselmis GSL1 HYDA transcripts that share very high similarity (99.5% nucleotide identity in the CDS), even higher in the promoter region. Moreover, the averaged abundance is very similar for both HYDA alleles (Table 2), suggesting no differential expression for the two allelic transcripts and consistent with a diploid organism.

Second, the Tetraselmis GSL1 transcriptome encodes two homologs to HYDE, one of which exists as a single transcript and the other of which exists as a HYDEF fusion. This represents the first report of the coexistence of 2 HYDE homologs in an algal genome, only one of which is fused inframe to HYDF. A single HYDEF fusion is observed in all other green algae with hydrogenase activity sequenced to date [25]. We speculate that the presence of two HYDE homologs in Tetraselmis GSL1 represents a key evolutionary intermediate pointing to the fusion event that led to the emergence of HYDEF, which may have occurred within the algal lineages as a way to economize protein translocation to the plastid. HYDE was putatively incorporated into algal genomes as a single gene early in the evolution of algae and can still be found as an independent form in Nannochloropsis where HYDE and HYDF remain separate despite being spatially located next to each other in a genomic hydrogenase gene cluster [71]. Analysis of the AA linker region between the HYDE and HYDF regions of fused HYDEF reveals no conserved motifs between currently sequenced algae (data not shown). If the fusion event occurred multiple times in various algal lineages this may account for the lack of any conserved HYDEF linker region because it was not present in the last common algal ancestor. Why Tetraselmis GSL1 retained an independent copy of HYDE is unknown; however, as shown in Table 2, the transcript abundances of HYDE and HYDEF are dramatically different (Table 2). This may be directly correlated to the function of HYDE as the putative source for the dithiolate ligand at the FeFe-hydrogenase active site [84], [85]; whereas, HYDF may function in a catalytic role, consistent with its role as a scaffold protein for active site construction and insertion [86]. It is unclear why HYDE would be fused to HYDF in this scenario.

Intriguing aspects of phylogenetically linked enzymatic adaptations are emerging among the various algal linkages and the sequences reported here provide additional evidence regarding the trajectory of algal hydrogenase acquisition and evolution. To date, eukaryotic algae have only been demonstrated to encode FeFe-hydrogenases [1] which is further supported by our transcriptome analysis. Inconclusive studies indicating the presence of NiFe-hydrogenases have been reported [87], [36]. Although the Bhosale et al. report contends that a NiFe-hydrogenase is present in a strain of Tetraselmis, we find no support of a NiFe-hydrogenase in Tetraselmis GSL1 and only find evidence of FeFe-hydrogenase. Very recently, a partial HYDA CDS from Tetraselmis subcordiformis was submitted to GenBank (Accession number: JQ317304), which has 85% similarity and 50% coverage with respect to the HYDA AA sequence reported here.

Among the Chlorophyceae, all FeFe-hydrogenases encode only the H-cluster binding domain and the presence of a hydrogenase with F-cluster binding domains has yet to be reported in this class. To date, these truncated H-cluster only hydrogenases are exclusively restricted to the Chlorophyceaen algae. It is unclear why these truncated variants emerged in evolution, but the presence of an H-cluster only enzyme in a halophilic specie of Tetraselmis (Chlorophyceae) is consistent with the presence of H-cluster only enzymes early in the evolution of the Chlorophyceae, and indicates that both fresh water and salt water members metabolically incorporate the H-cluster only enzyme variant. Currently, the most robust H_2_-photoproduction activity is observed in the Chlorophyceae and truncation may enable more efficient coupling to the photosynthetic electron transport chain [25]. There are now full-length algal FeFe-hydrogenase sequences for two algae outside of the Chlorophyceae [25], [71] and interestingly these organisms both encode enzymes with H-cluster and F-cluster domains and exhibit comparatively lower H_2_-photoproduction rates. The Trebouxiophyte, Chlorella variabilis NC64A contains two F-cluster FeFe-hydrogenases [25], whereas marine Nannochloropsis strains encode only a single F-cluster containing FeFe-hydrogenase [69].

Phylogenetic analyses of algal FeFe-hydrogenases demonstrate that the freshwater strains with sequenced transcriptomes/genomes contain at least two hydrogenase isoforms that are most similar to each other with respect to the other algal hydrogenases, indicating gene duplication within each of these individual algal lineages [25]. The marine organisms with available transcriptomes/genomes (Tetraselmis and Nannochloropsis [71], [88]) encode only a single HYDA isoform and both encode a copy of HYDE that is not fused to HYDF. Such observations may imply that marine organisms were not subject to the same evolutionary pressures that resulted in the hydrogenase duplication events observed in fresh water strains. The single HYDE protein is more difficult to interpret as Tetraselmis GSL1 also has HYDEF.

In sum, strains of Tetraselmis are promising candidates for the efficient production of H_2_ in saline systems and salt can be leveraged as a means to positively influence H_2_ production. The results of HYDA transcriptome sequencing and phylogenetic analyses are consistent with speculation that FeFe-hydrogenases emerged in algal genomes only a single time, perhaps as far back as the primary endosymbiotic event, and that only a single FeFe-hydrogenase with H-cluster and F-cluster domains was present [51], [25]. The FeFe-hydrogenase was likely a component of a suite of fermentation enzymes present in an ancestral eukaryote that functioned in adaptation to anoxia and was subsequently evolved to couple to the photosynthetic electron transport chain under some conditions. During evolution, the Chlorophyceae selected a truncated H-cluster only enzyme, and the green algae generated a HYDEF fusion by translocating the 5′-UTR of a single HYDF into the end of HYDE 3′-coding sequence to generate a new intron with in frame splicing to generate a HYDEF fusion. Freshwater algae then experienced selective pressures to duplicate the HYDA sequence, although for unknown reasons. Although additional research is required to better define and improve H_2_ production from halophilic species of Tetraselmis and new genome information is required to attain more definitive insights into the origins and evolution of hydrogenase in algae, the strains of Tetraselmis described here help to fill existing knowledge gaps in both of these areas.

## Supporting Information

Figure S1 **Schematic representation of the independent HYDE and fused HYDEF transcripts obtained from transcriptome sequencing.** The percent nucleotide identity of each transcript to each other is given. Detected SNPs between HYDE and HYDEF are also indicated. The insert shows a detailed view of the genomic differences (determined by PCR) between the two genes, specifically the loss of the stop codon and 3′UTR plus the addition of an intron in the HYDEF gene.
(PDF)

Table S1 **Hydrogenase activity comparisons between Tetraselmis strains and C. reinhardtii.** In vivo and in vitro activities of hydrogenase from halophilic strains of Tetraselmis and the fresh water alga C. reinhardtii are indicated.
(PDF)

Table S2 **Single nucleotide polymorphisms (SNPs) present in GSL1 HYD genes.** Detailed list of all SNPs found in HYD genes, nucleotide positions, allele frequencies and amino acid (AA) changes, if they occurred.
(PDF)
