# Supplementary material for: Evolutionary and Biotechnological Implications of Robust Hydrogenase Activity in Halophilic Strains of Tetraselmis
Source: PLoS One. 2014 Jan 21;9(1):e85812. doi: 10.1371/journal.pone.0085812 (PMC3897525; doi:10.1371/journal.pone.0085812)
Supplement: Figure S1 — Schematic representation of the independent HYDE and fused HYDEF transcripts obtained from transcriptome sequencing. The percent nucleotide identity of each transcript to each other is given. Detected SNPs between HYDE and HYDEF are also indicated. The insert shows a detailed view of the genomic differences (determined by PCR) between the two genes, specifically the loss of the stop codon and 3′UTR plus the addition of an intron in the HYDEF gene. (PDF) [file pone.0085812.s001.pdf]

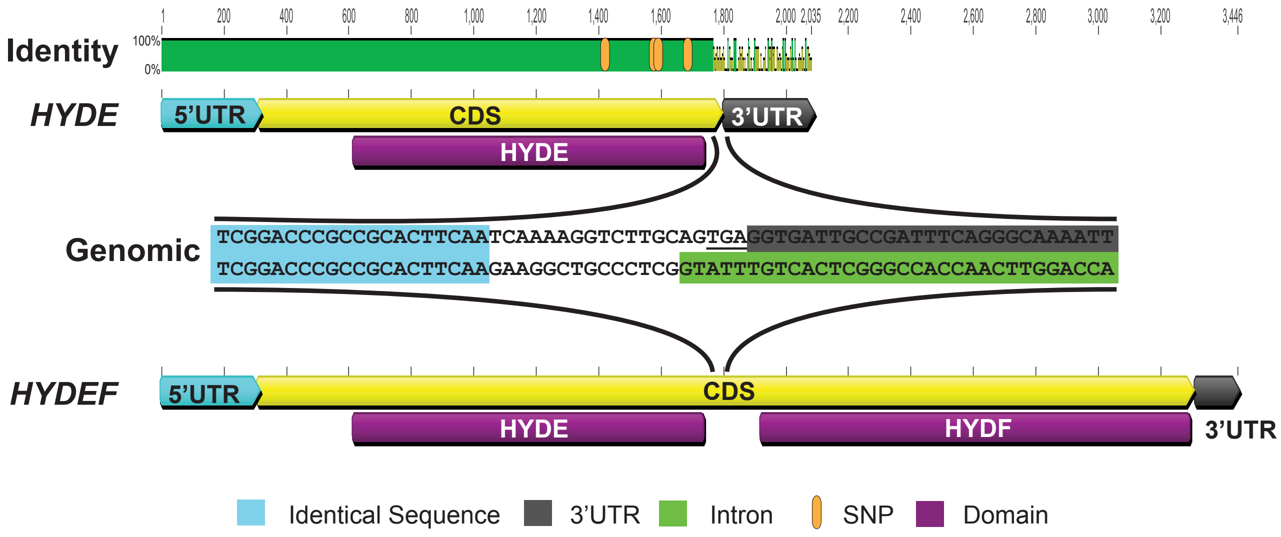

Figure S1. Schematic representation of the independent *HYDE* and fused *HYDEF* transcripts obtained from transcriptome sequencing.
