# Supplementary material for: Evolutionary and Biotechnological Implications of Robust Hydrogenase Activity in Halophilic Strains of Tetraselmis
Source: PLoS One. 2014 Jan 21;9(1):e85812. doi: 10.1371/journal.pone.0085812 (PMC3897525; doi:10.1371/journal.pone.0085812)
Supplement: Table S1 — Hydrogenase activity comparisons between Tetraselmis strains and C. reinhardtii. In vivo and in vitro activities of hydrogenase from halophilic strains of Tetraselmis and the fresh water alga C. reinhardtii are indicated. (PDF) [file pone.0085812.s002.pdf]

Table S1. Hydrogenase productivity comparisons between *Tetraselmis* strains and *C. reinhardtii*.

Hydrogen assay	*Tetraselmis* GSL1	*Tetraselmis* QNM1	*C. reinhardtii* 4h	*C. reinhardtii* 24h
**Photoproduction (nmol H_2•µg chl^{-1}•h^{-1})**	68.34 ± 2.46[a]	78.25 ± 0.89[a]	98 ± 5[b]	20 ± 10[b]
**Dark fermentative production (nmol H_2•µg chl^{-1})**	44.81 ± 4.06[a]	32.56 ± 4.38[a]	1.29 ± 0.56	1.93 ± 0.92
**Methyl-viologen (nmol H_2•µg chl^{-1}•h^{-1})**	**4h:**<br>1.6 % [NaCl][c]<br>700 ± 125<br>2.5 % [NaCl][c]<br>658 ± 148<br>3.18 % [NaCl][c]<br>534 ± 62	**4h:**<br>1.6 % [NaCl][c]<br>1064 ± 429<br>2.5 % [NaCl][c]<br>695 ± 207<br>3.18 % [NaCl][c]<br>608 ± 149	**4h:**<br>0 % [NaCl]<br>121 ± 27<br>1.6 % [NaCl][d]<br>129 ± 76<br>2.5 % [NaCl][d]<br>141 ± 57<br>3.18 % [NaCl][d]<br>146 ± 44	
	**24h:**<br>1.6 % [NaCl][c]<br>2190 ± 505<br>2.5 % [NaCl][c]<br>1476 ± 161<br>3.18 % [NaCl][c]<br>1260 ± 245	**24h:**<br>1.6 % [NaCl][c]<br>2035 ± 456<br>2.5 % [NaCl][c]<br>1766 ± 326<br>3.18 % [NaCl][c]<br>1734 ± 354		**24h:**<br>0 % [NaCl]<br>71 ± 13<br>1.6 % [NaCl][d]<br>65 ± 5<br>2.5 % [NaCl][d]<br>145 ± 86<br>3.18 %[NaCl][d]<br>117 ± 47
**Methyl-viologen (nmol H_2•µg tot $prot^{-1}$•h^{-1})**	**4h:**<br>1.6 % [NaCl][c]<br>14 ± 4.6<br>2.5 % [NaCl][c]<br>18.2 ± 7.9<br>3.18 % [NaCl][c]<br>15.5 ± 4.7	**4h:**<br>1.6 % [NaCl][c]<br>18.5 ± 4<br>2.5 % [NaCl][c]<br>14.2 ± 4.8<br>3.18 % [NaCl][c]<br>12.5 ± 64	**4h:**<br>0 % [NaCl]<br>2.9 ± 0.7<br>1.6 % [NaCl][d]<br>3.3 ± 1.9<br>2.5 % [NaCl][d]<br>3.4 ± 1.4<br>3.18 % [NaCl][d]<br>4 ± 2.3	
	**24h:**<br>1.6 % [NaCl][c]<br>56 ± 11.5<br>2.5 % [NaCl][c]<br>44 ± 4<br>3.18 % [NaCl][c]<br>37 ± 15	**24h:**<br>1.6 % [NaCl][c]<br>40 ± 16.7<br>2.5 % [NaCl][c]<br>37.6 ± 6<br>3.18 % [NaCl][c]<br>36 ± 6.8		**24h:**<br>0 % [NaCl]<br>1.7 ± 0.3<br>1.6 % [NaCl][d]<br>2 ± 1.4<br>2.5 % [NaCl][d]<br>3.3 ± 2<br>3.18% [NaCl][d]<br>2.8 ± 1

[a] 7.0% (w/v) NaCl, 24h anoxia induction.
[b] Data from Meuser, et al. (2012) [61].
[d] Anoxia induced in anaerobic induction buffer (AIB) and methyl-viologen (MV) assay performed in MV-f/2 solutions adjusted to the salinities indicated (final concentration). Values represent averages and standard deviations from 6 experimental repetitions.
