# Supplementary material for: Evolutionary and Biotechnological Implications of Robust Hydrogenase Activity in Halophilic Strains of Tetraselmis
Source: PLoS One. 2014 Jan 21;9(1):e85812. doi: 10.1371/journal.pone.0085812 (PMC3897525; doi:10.1371/journal.pone.0085812)
Supplement: Table S2 — Single nucleotide polymorphisms (SNPs) present in GSL1 HYD genes. Detailed list of all SNPs found in HYD genes, nucleotide positions, allele frequencies and amino acid (AA) changes, if they occurred. (PDF) [file pone.0085812.s003.pdf]

Table S2. Single nucleotide polymorphisms (SNPs) present in GSL1 *HYD* genes.

				Allele 1[b]			Allele 2[b]		
Gene[a]	SNP	Feature	Type	nt	Frequency[c]	AA[d]	nt	Frequency[c]	AA[d]
HYDA	M212	5'UTR	non-coding	C	22.9	-	A	75.4	-
HYDA	R222	CDS	non-synonymous	G	31.1	A2	A	68.8	T2
HYDA	Y291	CDS	non-synonymous	C	43.9	P25	T	54.6	S25
HYDA	Y331	CDS	non-synonymous	C	46.3	S38	T	52.7	L38
HYDA	Y343	CDS	non-synonymous	C	43.9	A42	T	53.4	V42
HYDA	W344	CDS	non-synonymous	T	43.3	A42	A	54.5	V42
HYDA	W389	CDS	synonymous	T	39.5	nc	A	55.7	nc
HYDA	W413	CDS	synonymous	A	42.4	nc	T	53.2	nc
HYDA	Y416	CDS	synonymous	T	43.3	nc	C	53.9	nc
HYDA	Y437	CDS	synonymous	T	44.0	nc	C	53.1	nc
HYDA	Y443	CDS	synonymous	C	49.1	nc	T	49.4	nc
HYDA	W464	CDS	synonymous	T	52.6	nc	A	44.6	nc
HYDA	M466	CDS	non-synonymous	C	55.0	A83	A	44.0	E83
HYDA	R467	CDS	non-synonymous	A	55.3	A83	G	43.9	E83
HYDA	Y680	CDS	synonymous	C	57.4	nc	T	41.7	nc
HYDA	Y683	CDS	synonymous	T	57.4	nc	C	42.2	nc
HYDA	S693	CDS	non-synonymous	C	57.5	P159	G	41.8	A159
HYDA	W713	CDS	synonymous	T	58.3	nc	A	40.1	nc
HYDA	R737	CDS	synonymous	G	58.2	nc	A	41.0	nc
HYDA	Y840	CDS	synonymous	C	50.2	nc	T	48.2	nc
HYDA	Y852	CDS	synonymous	T	48.8	nc	C	48.1	nc
HYDA	Y873	CDS	synonymous	C	52.8	nc	T	46.2	nc
HYDA	K882	CDS	synonymous	T	51.6	nc	G	48.1	nc
HYDA	Y909	CDS	synonymous	C	51.5	nc	T	47.0	nc
HYDA	R918	CDS	synonymous	A	49.1	nc	G	50.4	nc
HYDA	N1161	CDS	synonymous	A	53.1	nc	G	45.3	nc
HYDA	Y1183	CDS	synonymous	T	49.3	nc	C	47.3	nc
HYDA	W1321	CDS	synonymous	T	48.5	nc	A	49.2	nc
HYDA	M1345	CDS	synonymous	A	47.7	nc	C	47.6	nc
HYDA	Y1393	CDS	synonymous	T	49.6	nc	C	47.7	nc
HYDA	R1432	CDS	synonymous	G	56.9	nc	A	42.7	nc
HYDA	Y1465	CDS	synonymous	C	58.4	nc	T	40.5	nc
HYDA	Y1480	CDS	synonymous	T	58.1	nc	C	40.6	nc
HYDA	W1483	CDS	synonymous	T	59.1	nc	A	39.7	nc
HYDA	S1495	CDS	synonymous	C	59.2	nc	G	39.4	nc
HYDA	N1613	3' UTR	non-coding indel	-	NA	-	A	NA	-
HYDA	Y1679	3' UTR	non-coding	T	51.3	-	C	47.4	-
HYDA	K1703	3' UTR	non-coding	G	50.0	-	T	49.6	-
HYDA	Y1716	3' UTR	non-coding	T	51.2	-	C	48.0	-
HYDE/EF	R1419	CDS	synonymous	A	90.0	H370	G	8.5	R370

HYDE/EF	R1576	CDS	synonymous	G	86.7	nc	A	10.2	nc
HYDE/EF	Y1591	CDS	synonymous	T	90.5	nc	C	8.7	nc
HYDE/EF	K1685	CDS	synonymous	G	93.5	A459	T	5.9	S459
HYDE/EF	Y	INTRON	non-coding	C	80.6	-	T	13.1	-
HYDE/EF	R	INTRON	non-coding	A	85.7	-	G	14.3	-
HYDE/EF	Y	INTRON	non-coding	C	93.8	-	T	6.3	-
HYDE/EF	Y	INTRON	non-coding	T	88.3	-	C	10.6	-
HYDE/EF	Y	INTRON	non-coding	T	87.4	-	C	11.9	-
HYDE/EF	R	INTRON	non-coding	G	87.1	-	A	12.1	-
HYDE/EF	Y	INTRON	non-coding	C	85.1	-	T	14.5	-
HYDE/EF	Y	INTRON	non-coding	C	87.5	-	T	12.6	-
HYDE/EF	S	INTRON	non-coding	C	NA	-	G	NA	-
HYDE/EF	S	INTRON	non-coding	G	NA	-	C	NA	-
HYDE/EF	K	INTRON	non-coding	G	NA	-	T	NA	-
HYDE/EF	Y	INTRON	non-coding	C	NA	-	T	NA	-
HYDG	Y204	CDS	non-synonymous	C	49.7	R61	T	48.6	W61

[a] *HYDE/EF* indicates the comparison of the *HYDE* region of the *HYDE* and *HYDEF*.
[b] For *HYDE/EF* Allele 1 represents *HYDE* and Allele 2 represents *HYDEF*.
[c] Frequency, in percentage, of given allele found in sequenced transcriptome. NA- Frequency not applicable due to low coverage, type of SNP, or location in intron.
[d] AA for allele and location of AA in polypeptide. nc- no AA change.
